# A label-free fiber optic SPR biosensor for specific detection of C-reactive protein

**DOI:** 10.1038/s41598-017-17276-3

**Published:** 2017-12-04

**Authors:** Wenjia Wang, Zhigang Mai, Yuzhi Chen, Jiaqi Wang, Liang Li, Qingning Su, Xuejin Li, Xueming Hong

**Affiliations:** 1 College of Optoelectronic Engineering, Shenzhen University, Shenzhen, 518060 People’s Republic of China; 2 College of Physics and Energy, Shenzhen University, Shenzhen, 518060 People’s Republic of China; 3 School of Medicine,Shenzhen University, Shenzhen, 518060 People’s Republic of China; 4 Shenzhen Key Laboratory of Sensor Technology, Shenzhen, 518060 People’s Republic of China; 5 Shenzhen Engineering Laboratory for Optical Fiber Sensors and Networks, Shenzhen, 518060 People’s Republic of China

## Abstract

A highly sensitive and label-free fiber optic surface plasmon resonance (SPR) biosensor for specific detection of C-reactive protein (CRP) is proposed and demonstrated. We take dopamine as a cross-linking agent to immobilize the anti-CRP monoclonal antibody, which is an efficient and simple method for specific modification of the fiber optic SPR sensor. The modified sensor can successfully detect CRP specifically. We realize the fabrication of a disposable fiber optic SPR sensor for the CRP specific detection. Through optimizing the immobilization time of anti-CRP monoclonal antibody and the reaction time of antigen and antibody experimentally, the sensor shows a satisfactory linear response (R^2^ = 0.97) to CRP concentration within the range from 0.01 to 20 μg/ml. Moreover, the highest CRP sensitivity is obtained at 1.17 nm per lg (μg/ml). With the advantages of simple structure and easy fabrication, our sensor is convenient to be batch produced and controlled with good consistency, which is especially suitable for the fabrication of disposable biosensor. It makes sense that our detection can effectively avoid the cross pollution caused by repeated use of the sensor.

## Introduction

C-reactive protein (CRP) was initially reported by Tillett *et al*. in 1930, which was mainly synthesized by hepatocytes and used in response to infection, tissue inflammation or other inflammatory stimuli. More specifically, CRP is an acute phase response protein (APP) that can react with the polysaccharide of Streptococcus pneumoniae^1,2^. The concentration of CRP in the serum of a healthy person is below 5 µg/mL, and it always rises in the early stages of the antigen invasion. Therefore, many studies show that CRP is one of the most important inflammation markers in the human body^3^. In addition, it is found that CRP is helpful for the monitoring of exacerbations in chronic inflammatory conditions, including rheumatoid arthritis, inflammatory bowel disease, and a number of vasculitic syndromes^4^. Clinically, the CRP detection is usually used to differentiate the infection caused by bacteria from virus. Since CRP contributes importantly to the diagnosis of human diseases, a number of methods have been developed for its detection, such as enzyme linked immunosorbent assay (ELISA)^5^, immunofluorescence assay^6^ and latex agglutination^7^. However, these methods have the limitations of being time-consuming, semi-quantitative, and lack of miniaturization, and requirement of on-site analysis. In 2007, Chou C. employed fluorescence excitation based fiber optic biosensor for the CRP dectetion^8^, but the method is not label-free and with limited fluorescence collection efficiency. Recently, Zubiate P. presented a lossy mode resonance based optical fiber device for the selective CRP detection, which can detect concentration of CRP of 0.0625 mg/L^9^.

Different from the above detection methods, fiber optic surface plasmon resonance (SPR) sensing is a new biochemical detection method with the advantages of compact footprint, label-free detection, real-time monitoring, rapid detection and non-invasive measurements^10–12^. Generally, the biochemical reactions cause small environmental refractive index (RI) changes, while optic fiber SPR sensors are highly sensitive to these small RI changes. Detailed principle of fiber optic SPR sensing can be found in the Methods. Therefore, optic fiber SPR sensors have been widely used in various biosensing applications. Recently, Lu J. *et al*. reported a fiber optic SPR biosensor for determining infliximab concentrations in the serum from inflammatory bowel disease patients^13^. Their immunological sensor reached the detection limit of 0.3 ng/ml. In 2013, Tran D. T. *et al*. reported a fiber optic SPR DNA biosensor for the selection of DNA aptamers against the major peanut allergen protein, Ara h 1^14^. The selected aptamers specifically recognized Ara h 1 with a dissociation constant of 353 ± 82 nM. In 2015, Luo B. proposed a highly sensitive, label-free and selective fiber optic SPR glucose sensor^15^. Its detection concentration ranges from 0.1 to 2.5 mg/ml with a sensitivity of ~1.514 nm (mg/ml)^−1^. Recently, Aray A. *et al*. proposed a SPR-based plastic optical fiber biosensor for the detection of CRP in serum^16^. Their sensor worked in transmission type which lacks flexibility and portability compared with the reflection type. Moreover, most of the optic fiber SPR biosensors, as mentioned above, are reused in a variety of detections, which may lead to the cross contamination in the detections or bring errors in the practical applications.

In this paper, we propose and demonstrate a label-free fiber optic SPR biosensor for specific detection of CRP concentration. We realize the fabrication of disposable fiber optic SPR sensor for the CRP specific detection. The fiber sensor is prepared by depositing an Au film on the sensing region of an unclad multi-mode silica fiber, while its sensitivity is evaluated by measuring NaCl solutions with different concentrations. Detailed fabrication process of the fiber optic SPR sensor and experimental setup can be found in the Methods. In the process of biological modification and detection, the optic fiber SPR biosensor is firstly modified with a biological crosslinked membrane (polydopamine (PDA)) by the surface functionalization method as reported in the previous literature^17^. Afterward, we immobilize the anti-CRP monoclonal antibody on the sensor surface for the CPR specific detection. Detailed process of biological modification and specific detection can be found in the Methods. Finally, the CPR specific detection ability of the modified sensor is investigated. Our simple and compact device is promising for mass production with well-controlled consistency, which opens up the possibility of using the applications in disposable biochemical detections.

## Results and Discussion

### Performance evaluation of the fiber optic SPR sensor

**Figure 1:**
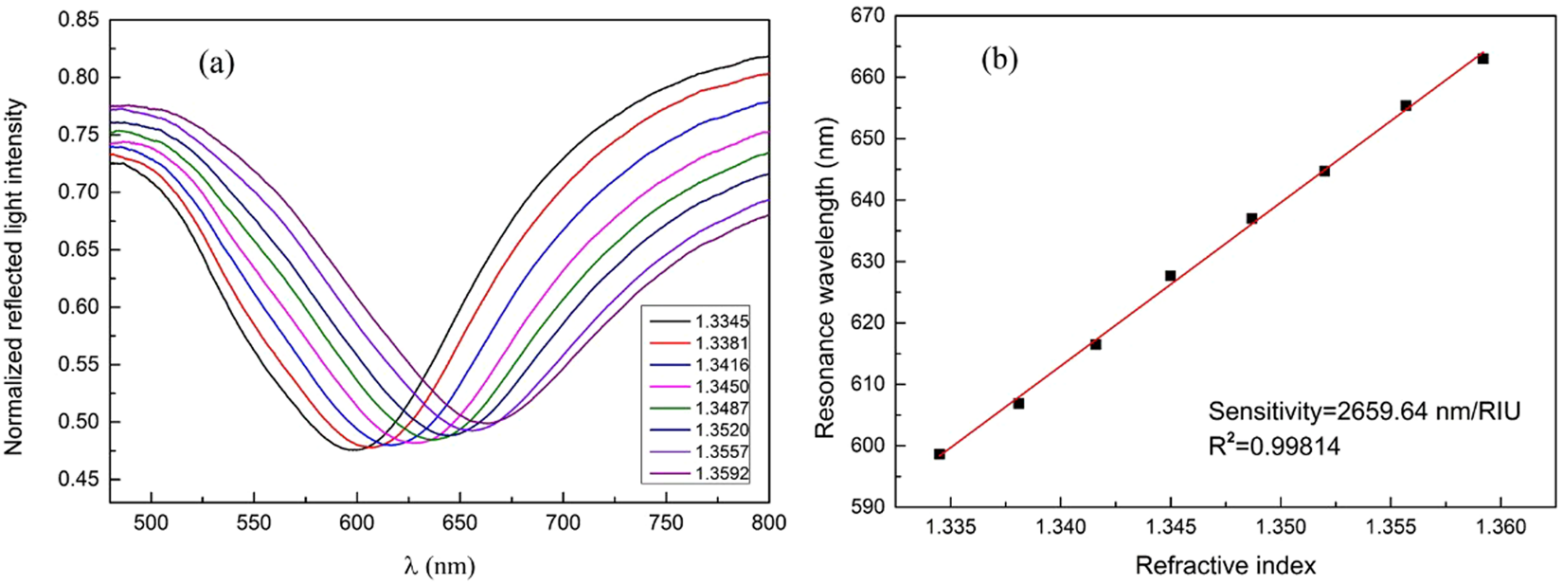
Performance evaluation of the fiber optic SPR sensor. (**a**) Normalized reflection spectra in measuring NaCl solutions with different RI values. (**b**) Experimental measurement of the external RI-dependent resonance wavelengths.

Figure 1(a) shows normalized reflection spectra of the fiber optic SPR sensor in measuring NaCl solutions with different concentrations. The SPR dip shifts towards longer wavelengths with the increase of RI, and the response of the resonance wavelength shows a linear relation with the RI change, as shown in Fig. 1(b). The sensitivity of the sensor is 2659.64 nm/RIU for RI ranging from 1.3345 to 1.3592, which is sufficiently high for biosensing purposes. Detailed sensitivity test of the fiber optic SPR sensor can be found in the Methods. In addition, in order to examine the uniformity of our sensors, all the sensors with the same preparation parameters are examined by measuring deionized water and ethanol. Considering that the relative standard deviation of the resonance wavelength differences in measuring deionized water and ethanol is 1.6%, our sensors have a good consistency.

### Comparison of specific and nonspecific detections

**Figure 2:**
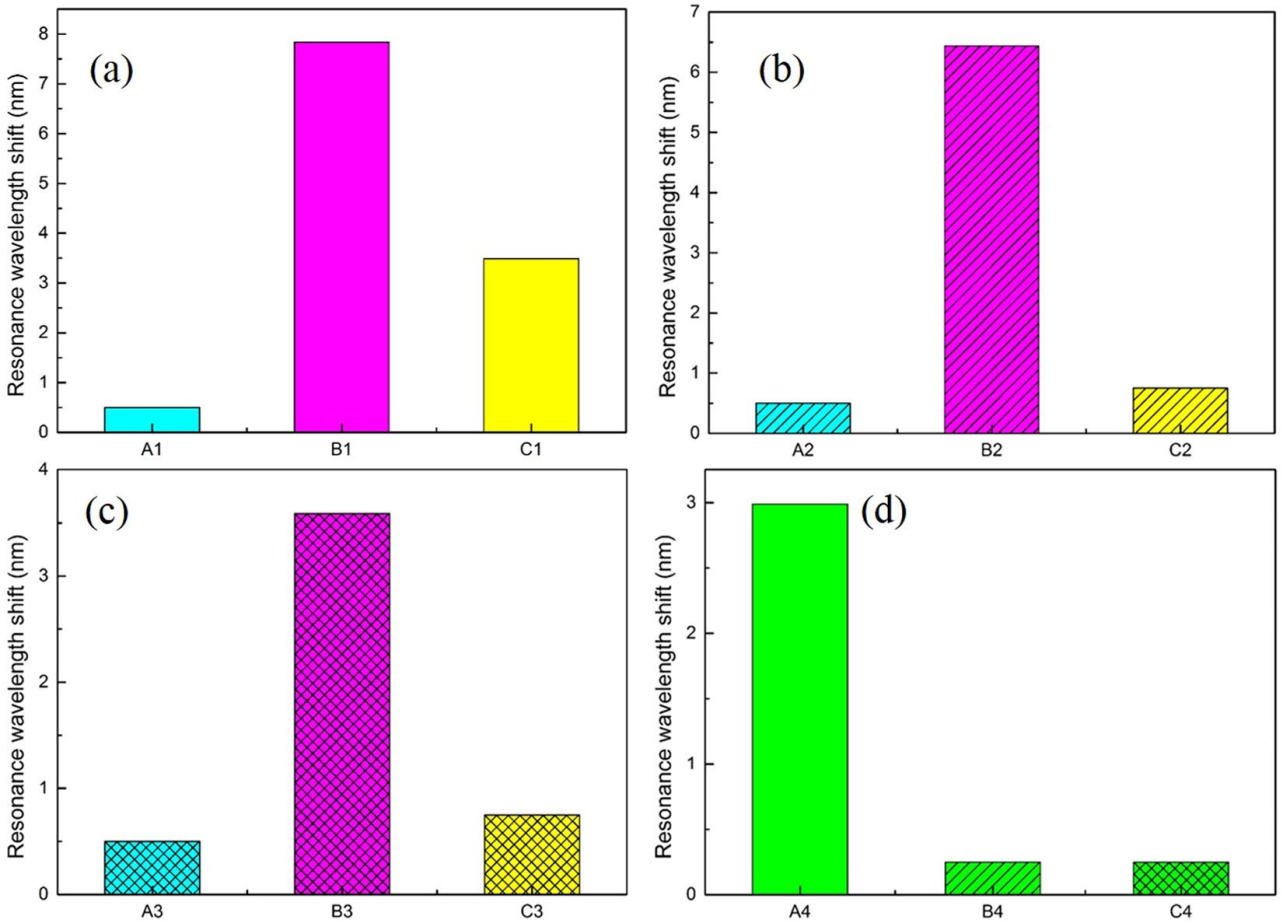
Comparison of specific and nonspecific detection of the modified fiber optic SPR sensors (All the letters under the abscissa of (**a**–**c**) represent the detection environments which the sensors are located in). (**a**) Anti-CRP monoclonal antibody immobilized sensor for the specific detection of CRP. A1: PBS buffer, B1: CRP, C1: PBS buffer after antigen-antibody binding. (**b**) Eab monoclonal antibody immobilized sensor for the nonspecific detection of CRP. A2: PBS buffer, B2: CRP, C2: PBS buffer after antigen-antibody binding. (**c**) Anti-CRP monoclonal antibody immobilized sensor for the nonspecific detection of BSA. A3: PBS buffer, B3: BSA, C3: PBS buffer after antigen-antibody binding. (**d**) Comparison of the resonance wavelength differences before and after the detections of specificity and nonspecificity. A4: the resonance wavelength difference of C1 and A1, B4: the resonance wavelength difference of C2 and A2, C4: the resonance wavelength difference of C3 and A3.

The specificity of the CRP detection is analyzed from two aspects. In one aspect, the specific target detection ability is analyzed through comparatively testing the CRP and bovin serum albumin (BSA) solutions with the same concentration by the anti-CRP monoclonal antibody immobilized sensor. The main steps are as follows. First, the PDA functionalized sensors are immersed in 50 μg/ml solution of anti-CRP monoclonal antibody diluted with phosphate buffered saline (PBS) buffer for 4 hours. Second, the anti-CRP monoclonal antibody immobilized sensors are used for detecting 50 μg/ml solutions of CRP and BSA diluted in PBS buffer, respectively. At last, the sensors in the second step are immersed in PBS buffer again. The shifts of their resonance wavelengths of the two sensors in each of the three steps are shown in Fig. 2(a) and 2(c), respectively.

In the other aspect, the specific modifications of the sensor are analyzed. The detection of CRP is comparatively tested between the anti-CRP monoclonal antibody immobilized sensor and the other non-anti-CRP monoclonal antibody immobilized sensor. Following are the main steps. The PDA functionalized sensors are immersed in 50 μg/ml solutions of anti-CRP monoclonal antibody and Eab antibody (the antibody with no response to CRP) diluted with PBS buffer for 4 hours, respectively. Then, the anti-CRP monoclonal antibody immobilized sensor and Eab antibody immobilized sensor are used for detecting 50 μg/ml solution of CRP diluted in PBS buffer, respectively. Finally, the two sensors in the second step are immersed in PBS again. The shifts of their resonance wavelengths of the two sensors in each of the three steps are shown in Fig. 2(a) and 2(b).

To avoid the concentration effect of different solutions on the SPR resonance shift, we use the wavelength shift difference in the first step and the third step as the criterion to evaluate the specific and nonspecific detection, as shown in Fig. 2(d). From the detection results of the two aspects, it reveals that the resonance wavelength shift of specific detection is 2.99 nm (obvious effect), while the resonance wavelength shift of nonspecific detection is about 0.25 nm (non-obvious effect). Therefore, experimental results show that our anti-CRP monoclonal antibody immobilized sensor has the ability of CRP specific detection.

### Optimal immobilization time for anti-CRP monoclonal antibody

**Figure 3:**
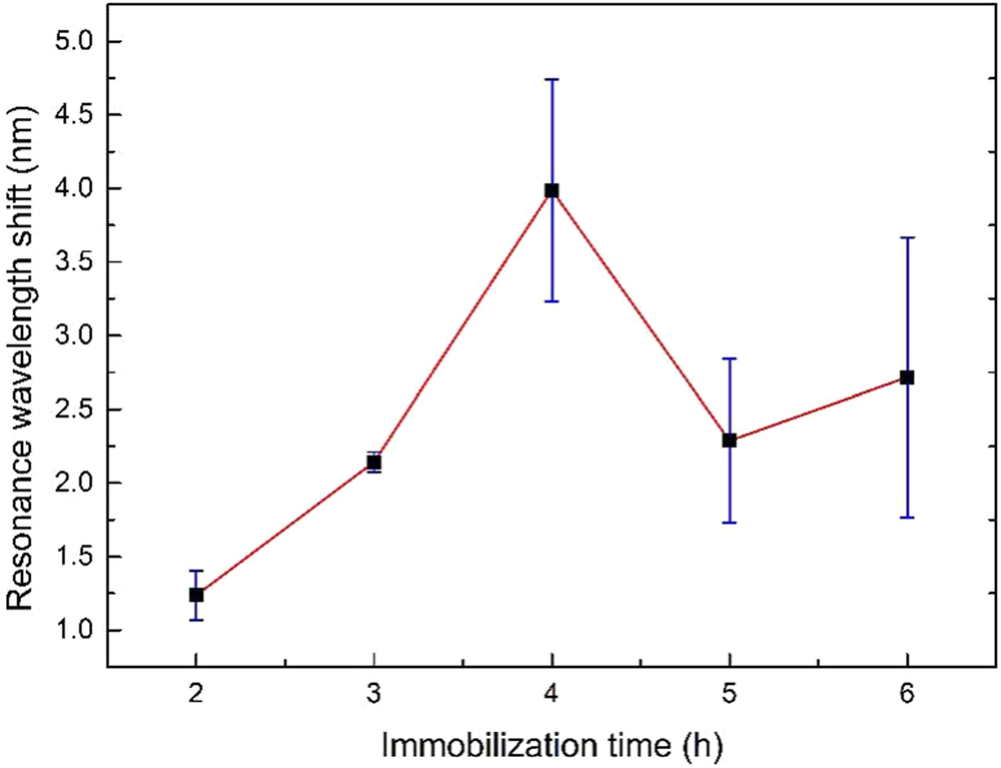
The resonance wavelength shift as a function of the immobilization time of anti-CRP monoclonal antibody.

To achieve the satisfactory performance of our sensor, the effective immobilization of the anti-CRP monoclonal antibody on the PDA functionalized sensor is crucial in the CRP detection. We investigate the best immobilization time of the anti-CRP monoclonal antibody. After being functionalized with a fine PDA layer, several sensors are immersed in the anti-CRP monoclonal antibody solution (50 μg/ml diluted with PBS buffer) for 2 hours, 3 hours, 4 hours, 5 hours and 6 hours, respectively, to immobilize with anti-CRP monoclonal antibody layers. We use the immobilized sensors to detect 50 μg/ml solutions of CRP diluted in PBS buffer for the selection of optimal immobilization time. The selection criterion is based on the resonance wavelength shift before and after the CRP detection (the larger shift, the better). The maximum resonance wavelength shift for the antibody immobilization is 3.99 nm at 4 hours, and the corresponding standard deviation of each experimental point (Repeated 3 times disposable detections) is also detailed in Fig. 3 as blue error bars. The resonance wavelength shift indicates that the number of antibodies binding to the sensor surface is increasing within 4 hours. After that, the resonance wavelength shift is smaller than that observed at 4 hours. The optimal CRP detection result of the immobilized sensors is also at 4 hours. Thus, the most efficient immobilization efficiency of the anti-CRP monoclonal antibody is 4 hours considering the overall change trend and time cost, which is chosen in all further experiments.

### Reaction time and sensitivity of CRP detection

**Figure 4:**
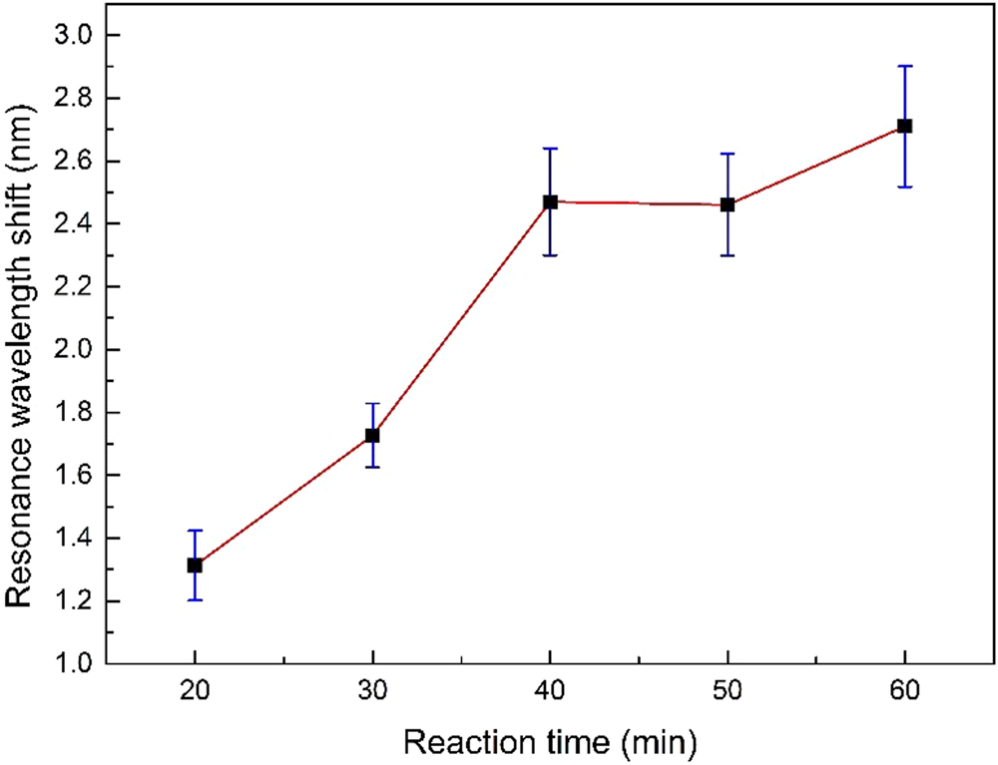
The resonance wavelength shift as a function of reaction time of CRP with the anti-CRP monoclonal antibody immobilized sensor.

In order to achieve the best performance for the CRP detection, the reaction time of CRP with our anti-CRP monoclonal antibody immobilized sensor must be optimized at first. We put the anti-CRP monoclonal antibody immobilized sensors into 50 μg/ml solutions of CRP diluted in PBS buffer and record the resonance wavelength shifts after 20 mins, 30 mins, 40 mins, 50 mins and 60 mins, respectively. The resonance wavelength shifts for different reaction times are shown in Fig. 4 which is along with corresponding error bar at each point. It is obtained that the maximum error bar of the measured wavelength shifts is ±0.19 nm, which indicates that the sensor has high detection repeatability. The resonance wavelength shift increases rapidly in the first 40 mins due to a fast reaction rate, while it has only slightly improved with additional 0.24 nm at 60 mins. This shows that between 40 mins and 60 mins, the reaction process gradually reaches the equilibrium condition. Considering the stability of reaction and clinical purpose, the resonance wavelength shift is recorded at 60 mins in the further experiment.

To evaluate the sensitivity of the detection of CRP, different concentrations (0.01 μg/ml, 0.1 μg/ml, 1.0 μg/ml, 10 μg/ml, 20 μg/ml) of CRP solutions are detected. According to the normal concentration of protein in a health person is 6%–8%, another set of control experiments with different concentrations of BSA (untargeted protein) have been carried. Their concentrations are 1%, 2%, 5%, 8%, 10%, corresponding to 10 mg/ml, 20 mg/ml, 50 mg/ml, 80 mg/ml, 100 mg/ml, respectively.

**Figure 5:**
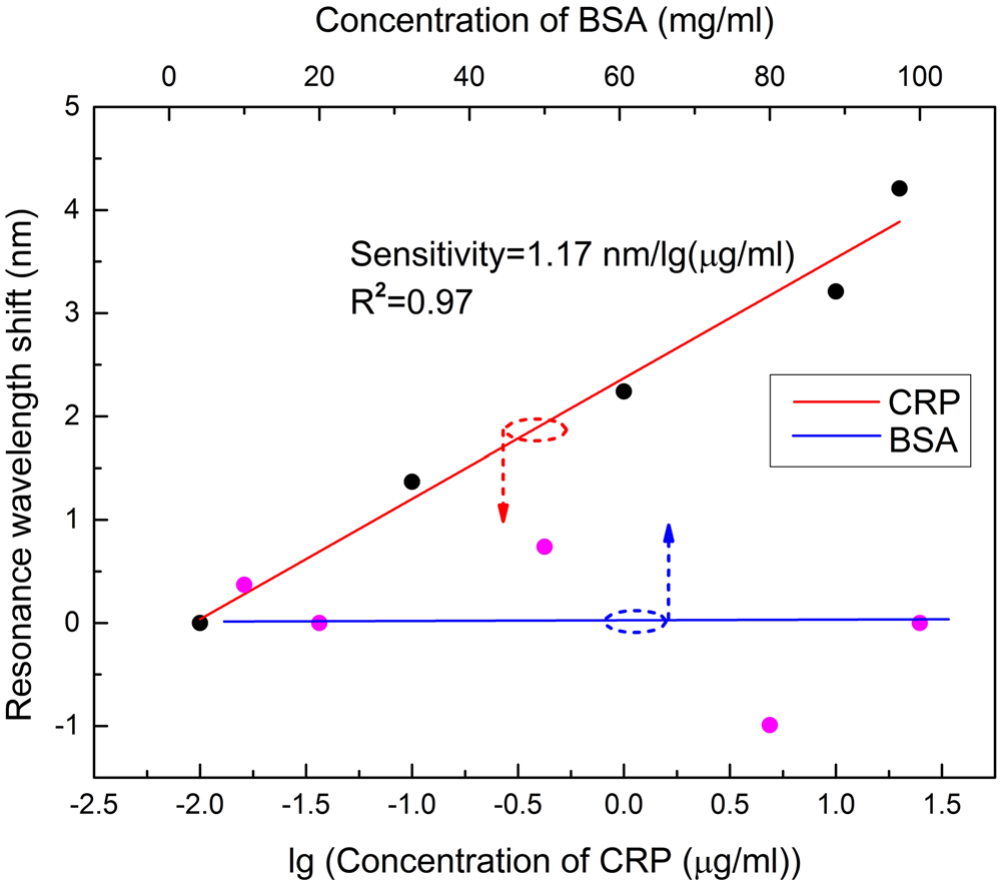
The resonance wavelength shift as a function of the logarithm concentration of CRP (in red colour) and concentration of BSA (in blue colour).

The relationship between CRP concentration or BSA concentration and resonance wavelength shift is shown in Fig. 5. It is observed that the resonance wavelength shift increases with the CRP concentration, while the resonance wavelength shift is slightly fluctuated in the BSA detections with the concentrations ranging from 10 mg/ml to 100 mg/ml. The anti-CRP monoclonal antibody immobilized sensor for BSA detection is non-obvious effect due to the nonspecific detection, and it is confirmed that this is not a dilute concentration effect. Moreover, our sensor exhibits a satisfactory linear response (R^2^ = 0.97) to the logarithm of CRP concentration ranging from 0.01 to 20 μg/ml. The maximum difference of the resonance wavelength shift is found to be around 4.21 nm. Sensitivity, defined as the shift of the resonance wavelength per unit change in the logarithm concentration of CRP, is calculated to be 1.17 nm per lg (μg/ml). Experimental results reveal that the resonance wavelength shift increases with the increasing concentration of CRP. And in this range, the resonance wavelength shift is proportional to the CRP concentration.

## Methods

### Principle of fiber optic SPR sensing

**Figure 6:**
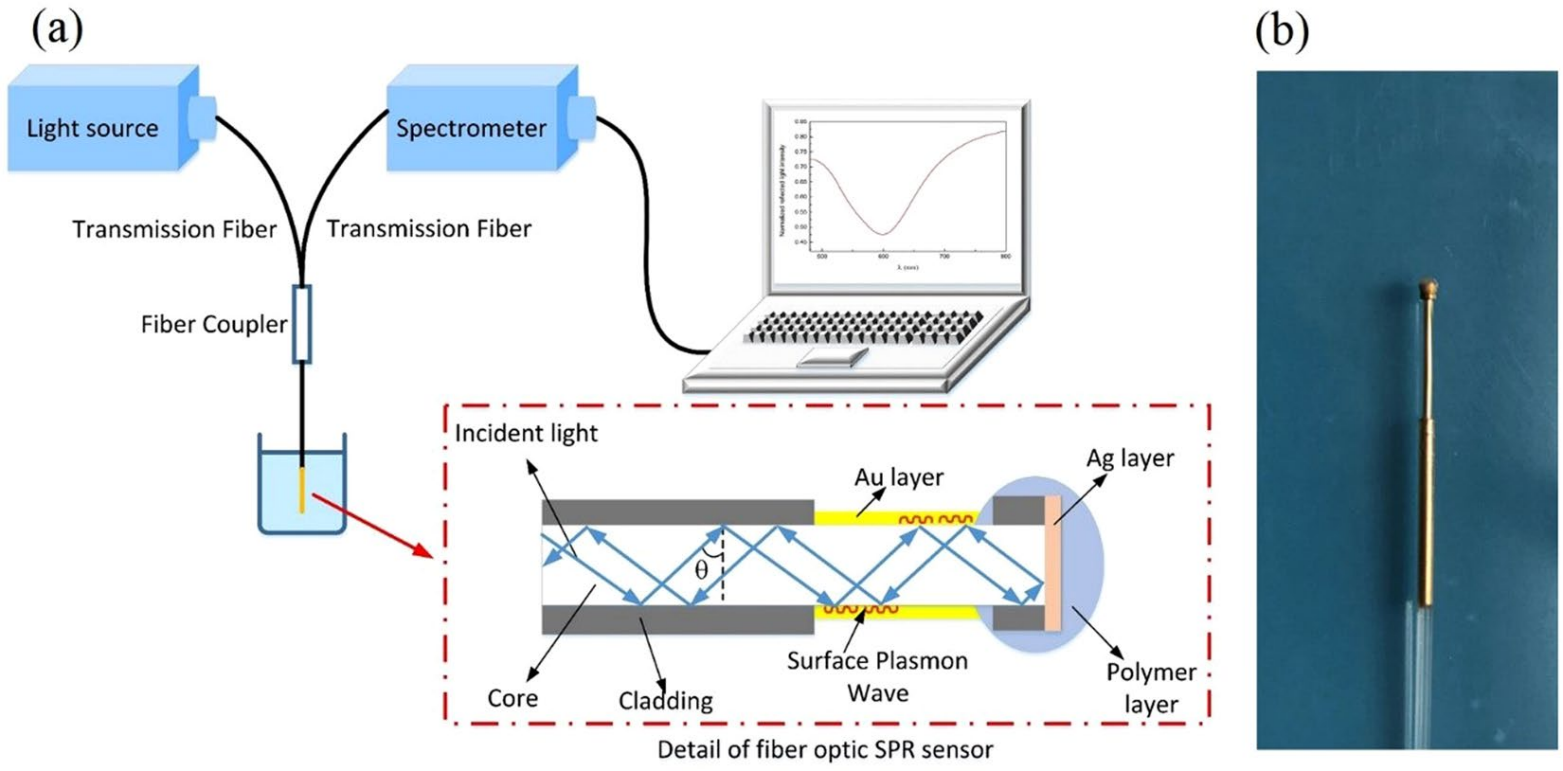
Fiber optic SPR sensing system. (**a**) Schematic diagram of experimental setup; (**b**) Image of the fabricated fiber optic SPR sensor.

In general, SPR is excited at the interface between metal and dielectric by the evanescent field of the optical wave. For the fiber optic SPR sensor, a multi-mode fiber is used as the optical waveguide medium, while a nanoscale metal film is deposited on the sensing region in order to excite plasma. The model of fiber optic SPR sensor is illustrated in Fig. 6(a). When the incident light propagates through the fiber core on the condition that the incident angle is greater than the critical angle, the evanescent field generated from total reflection of the incident light will reveal into the metal film simultaneously. When the parallel component of the incident light wave vector matches that of the surface plasmon wave, a strong absorption of light occurs. As a result, a SPR dip at a particular wavelength, which is known as resonance wavelength, appears in the output signal. As detailed in previous papers^18–21^, the transmitted power of the SPR spectrum is given by:

1 $${P}_{trans}=\frac{{\int }_{{\theta }_{cr}}^{\pi /2}({R}_{p}^{N(\theta )}+{R}_{s}^{N(\theta )})P(\theta )d\theta }{{\int }_{{\theta }_{cr}}^{\pi /2}P(\theta )d\theta }$$

where *N*(θ) = 2 *L*/dtanθ, θ_cr_ = sin^−1^(n_cl_/n_1_), P(θ) = n_1_^2^sinθcosθ/(1− n_1_^2^cos^2^θ)^2^. N(θ) denotes the number of the reflections performed by the light with an incident angle θ in the sensing area, while *L* and d are the length of sensing region and the fiber core diameter, respectively. θ_cr_ is the critical angle of the incident light in the fiber, n_cl_ and n_1_ are the refractive index of the fiber cladding and the fiber core, respectively. P(θ) is the modal power as a function of the incident angle θ. R_p_ and R_s_ define the reflection ratio of p- and s-polarized light. Only p-polarized light can generate surface plasma, while s-polarized light makes no contribution to SPR.

### Fabrication of the fiber optic SPR sensor

The fiber optic SPR sensor is fabricated on a multi-mode plastic clad silica fiber (Nanjing Chunhui Inc.) with the core diameter of 600 μm and the numerical aperture of 0.37. At one end of the fiber, we choose an appropriate region and remove its coating and cladding with the length of 5 mm by a sharp blade. In order to fabricate a terminal reflective sensor, the end face of the sensor should be fully polished by 4 kinds of lapping films with different roughness (30, 9, 3, and 1 μm, LUSTER Inc.). Afterwards, the unclad part and the end face of the sensor are cleaned with acetone solution and deionized water three times. Finally, an Au sensing layer with 50 nm thickness is deposited on the unclad part of the sensor, while an Ag reflector with 300 nm thickness is deposited on the end face of the sensor by using the magnetron sputtering technology. It is worth to mention, in order to prevent the Ag reflector from being oxidized in various biochemical solutions, the sensor end with Ag reflector should be effectively protected by a polymer covering layer (Modified Acrylate Adhesive). The image of the fiber optic SPR sensor is shown in Fig. 6(b).

### Experimental setup

The schematic diagram of the sensing system is shown in Fig. 6(a). The white light from the halogen source (Ocean Optics Inc., DH-2000-BAL) firstly transmits into the fiber optic SPR sensor through the transmission fiber (Thorlabs Inc., multi-mode fiber with 600 μm core) and coupler. Then the white light is modulated by external environmental solutions in the sensing region when the sensor is immersed into the target solution. Finally, the modulated light is reflected back by the Ag reflector and collected by a USB 4000 spectrometer (Ocean Optics Inc., 200–1100 nm). After a normalization process (detailed in our previous study^22^), the SPR spectrum is obtained. Our normalization process can dynamically detect the lowest point of the SPR dip and effectively eliminate the background noise.

### Sensitivity test of the fiber optic SPR sensor

Since the SPR phenomenon is very sensitive to the RI changes near the surface of Au layer, and the CRP specific detection would inevitably cause the surrounding RI changes of the fiber optic SPR sensor. The RI sensitivity of the sensor before modification should be evaluated. The RI sensitivity is experimentally determined by measuring a set of NaCl solutions with concentrations ranging from 0 to 14%. Their RI, ranges from 1.3345 to 1.3592, are calibrated by WYA-2S digital Abbe refractometer at the room temperature.

### Materials of biological modification and detection

Dopamine hydrochloride and bovin serum albumin (BSA) are purchased from Sigma-Aldrich. Anti-CRP monoclonal antibody and Eab are purchased from Proteintech Co. Phosphate buffered saline (PBS, 10 mM, pH 7.4, 1.76 mM KH_2_PO_4_, 10.14 mM Na_2_HPO_4_·12H_2_O, 136.75 mM NaCl, 2.68 mM KCl) and Tris-HCl buffer (10 mM, pH 8.5) are prepared with ultra-pure water. All the chemicals used are of analytical reagent grade.

### Process of biological modification and specific detection

**Figure 7:**
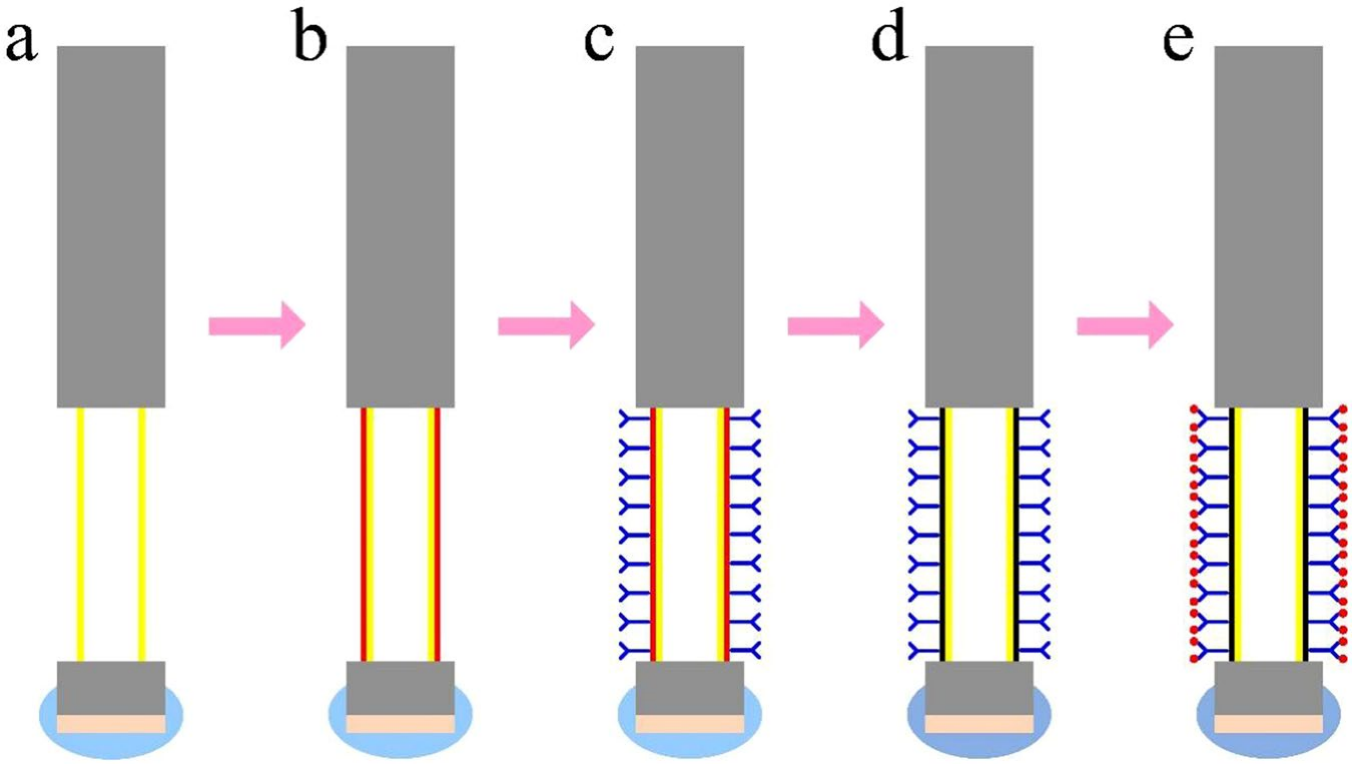
Steps for the biological modification and specific detection of the fiber optic SPR sensor for the CRP detection: (**a**) Non-loaded sensor; (**b**) Biological crosslinking of dopamine on the surface of the non-loaded sensor; (**c**) Immobilization of anti-CRP monoclonal antibody on the PDA modified sensor; (**d**) PDA oxidation of the specific modified sensor; (**e**) CRP detection of the specific modified sensor.

**Figure 8:**
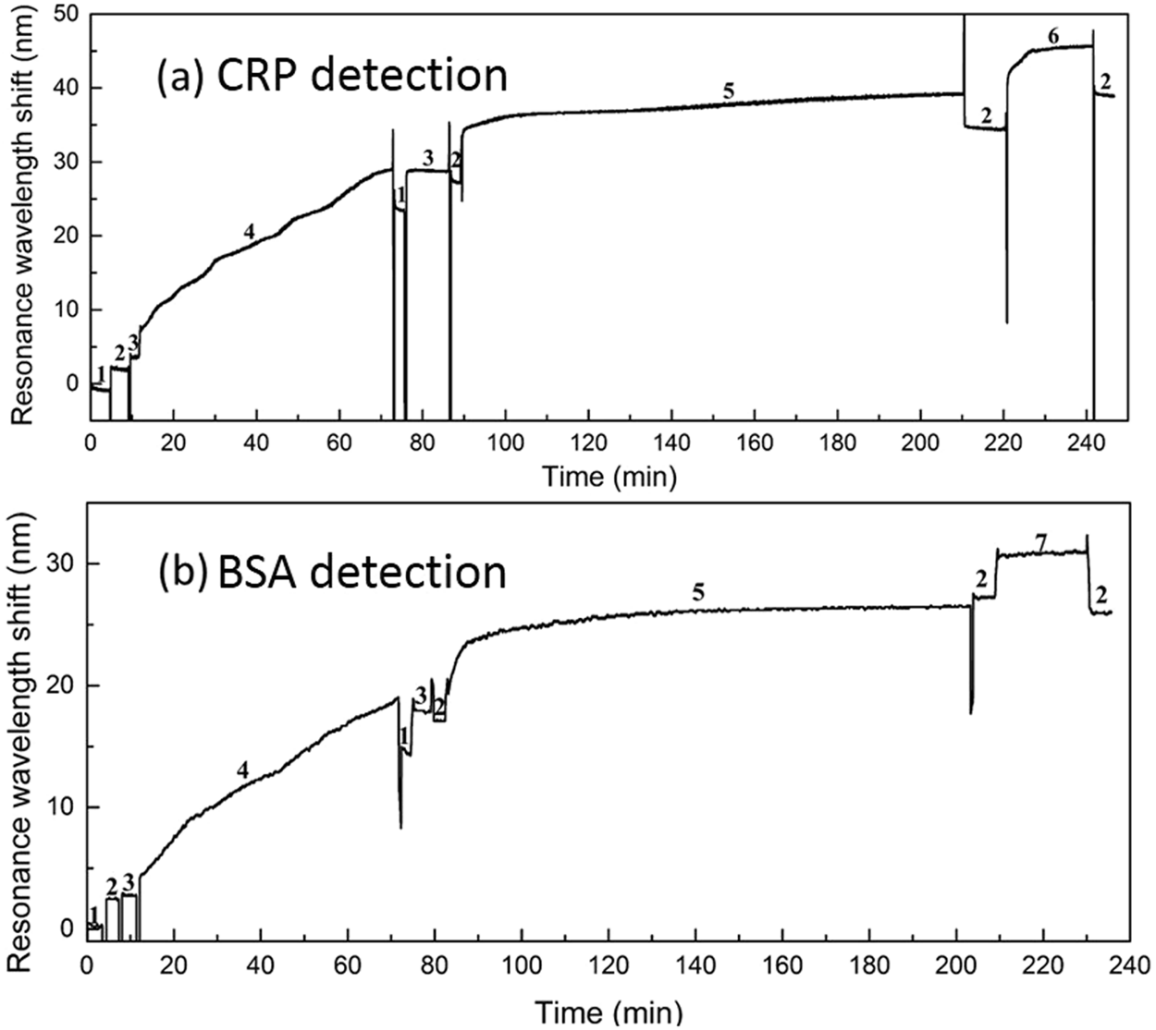
The SPR resonance wavelength shift monitoring during the biological modification and detection process (**a**) specific detection of CRP; (**b**) nonspecific detection of BSA. Both the CRP and BSA concentration used are 50 μg/ml. Numbers represent the solutions which the sensor is located in: 1. deionized water, 2. PBS buffer, 3. Tris-HCl buffer, 4. dopamine, 5. monoclonal anti-CRP antibody, 6. CRP, 7. BSA.

The schematic of the biological modification and specific detection processes of the fiber optic SPR sensor is shown in Fig. 7. The detailed procedure is as follows. After cleaning with deionized water, the prepared sensor is immersed in PBS buffer for the adaptation of the specific reaction environment. Then the sensor surface is functionalized with a PDA film by immersed in a 2.0 mg/ml dopamine solution (dopamine dissolved in Tris-HCl buffer) for 60 min. After washed with deionized water and Tris-HCl buffer, the sensor is immersed in the anti-CRP monoclonal antibody solution (solvent is PBS buffer). Afterwards, the anti-CRP monoclonal antibodies are immobilized on the surface of PDA functionalized sensor by covalent binding. When the adsorption equilibrium is reached, we put the sensor into PBS buffer to remove the antibodies that are bound to its surface. Then the antibody immobilized sensor is ready for detecting CRP. In order to effectively avoid cross contamination, each sensor is used for the disposable detection. In the experiment, all the steps are operated at room temperature. Figure 8(a) records an example of the SPR resonance wavelength shift during the process of biological modification and specific detection, while control data are displayed with the real-time follow up of the SPR response measured in presence of BSA after the full optic fiber functionalization as shown in Fig. 8(b). As shown in Fig. 8(a), there is a typical one-step immunoassay in this example, and the reaction of antigen and antibody can be monitor by our SPR biosensor system. The amount of antigen and antibody response can also be indicated by the resonance wavelength shift. Furthermore, it can be observed that the resonance wavelength shift difference in BSA detection is much smaller than that of CRP detection obviously from Fig. 8, resulting from the nonspecific and specific detections.

## References

[1] Tillett WS, Francis T (1930). Serological reactions in pneumonia with a non-protein somatic fraction of pneumococcus. The J Exp. Med..

[2] Macleod CM, Avery OT (1941). The occurrence during acute infections of a protein not normally present in the blood: II. Isolation and properties of the reactive protein. The J Exp. Med..

[3] Clyne B, Olshaker JS (1999). The C-reactive protein. The J Emerg. Med..

[4] Young B, Gleeson M, Cripps AW (1991). C-reactive protein: a critical review. Pathol..

[5] Barka N, Tomasi JP, Stadtsbaeder S (1985). Use of whole Streptococcus pneumoniae cells as a solid phase sorbent for C-reactive protein measurement by ELISA. J Immunol. Methods.

[6] Harma H, Toivonen J, Soini JT (2011). Time-resolved fluorescence immunoassay for C-reactive protein using colloidal semiconducting nanoparticles. Sensors.

[7] Senju O, Takagi Y, Uzawa R (1986). A new immuno quantitative method by latex agglutination–application for the determination of serum C-reactive protein (CRP) and its clinical significance. J Clin. Lab Immunol..

[8] Chou C, Hsu H-Y, Wu H-T (2007). Fiber optic biosensor for the detection of C-reactive protein and the study of protein binding kinetics. J. Biomed. Opt..

[9] Zubiate P, Zamarreño CR, Sánchez P, Matias IR, Arregui FJ (2017). High sensitive and selective C-reactive protein detection by means of lossy mode resonance based optical fiber devices. Biosens. Bioelectron..

[10] Shankaran DR, Gobi KV, Miura N (2007). Recent advancements in surface plasmon resonance immunosensors for detection of small molecules of biomedical, food and environmental interest. Sens. Actuator B.

[11] Huang H, Chen Y (2006). Label-free reading of microarray-based proteins with high throughput surface plasmon resonance imaging. Biosens. Bioelectron..

[12] Gobi KV, Miura N (2004). Highly sensitive and interference-free simultaneous detection of two polycyclic aromatic hydrocarbons at parts-per-trillion levels using a surface plasmon resonance immunosensor. Sens. Actuator B.

[13] Lu J, Stappen TV, Spasic D (2016). Fiber optic-SPR platform for fast and sensitive in fliximab detection in serum of inflammatory bowel disease patients. Biosens. Bioelectron..

[14] Tran DT, Knez K, Janssen KP (2013). Selection of aptamers against Ara h 1 protein for FO-SPR biosensing of peanut allergens in food matrices. Biosens. Bioelectron..

[15] Luo B, Yan Z, Sun Z (2015). Biosensor based on excessively tilted fiber grating in thin-cladding optical fiber for sensitive and selective detection of low glucose concentration. Opt. Express.

[16] Aray A, Chiavaioli F, Arjmand M (2016). SPR-based plastic optical fibre biosensor for the detection of C-reactive protein in serum. J. Biophotonics.

[17] Shi S, Wang L, Su R (2015). A polydopamine-modified optical fiber SPR biosensor using electro-less-plated gold films for immunoassays. Biosens. Bioelectron..

[18] Chen YZ, Yu YQ, Li XJ (2016). Fiber-optic urine specific gravity sensor based on surface plasmon resonance. Sens. Actuator B.

[19] Liu BH, Jiang YX, Zhu XS (2013). Hollow fiber surface plasmon resonance sensor for the detection of liquid with high refractive index. Opt. Express.

[20] Perrrotton C, Javahiraly N, Slaman M (2011). Fiber optic surface plasmon resonance sensor based on wavelength modulation for hydrogen sensing. Opt. Express.

[21] Yuan YQ, Ding LY, Guo ZQ (2011). Numerical investigation for SPR-based optical fiber sensor. Sens. Actuators B: Chem..

[22] Tan ZX, Li XJ, Chen YZ, Fan P (2013). Real-time measurement with a fiber opticalsurface plasmon resonance sensor for biochemical interaction analysis. J. Proc.SPIE.

